# Editorial: A Hippo's View: From Molecular Basis to Translational Medicine

**DOI:** 10.3389/fcell.2021.729155

**Published:** 2021-07-23

**Authors:** Zhaocai Zhou, Zengqiang Yuan, Wanjin Hong, Wenqi Wang

**Affiliations:** ^1^State Key Laboratory of Genetic Engineering, Zhongshan Hospital, School of Life Sciences, Fudan University, Shanghai, China; ^2^The Brain Science Center, Beijing Institute of Basic Medical Sciences, Beijing, China; ^3^Institute of Molecular and Cell Biology, Agency for Science, Technology, and Research (A*STAR), Singapore, Singapore; ^4^Department of Developmental and Cell Biology, University of California, Irvine, Irvine, CA, United States

**Keywords:** Hippo pathway, organ size control, YAP, TAZ, cancer

Initially identified in *Drosophila*, the major components of the Hippo pathway including the core kinase cascade, downstream effector and nuclear transcription factor are evolutionarily conserved, resulting in extensive studies from many investigators in the past decades. The Hippo pathway is now recognized as a key player in organ size control *via* primarily stimulating programmed cell death and restricting cell proliferation (Halder and Johnson, 2011; Yu et al., 2015; Zheng and Pan, 2019).

The Hippo pathway regulates cell proliferation, survival and differentiation in response to a wide range of extracellular cues including growth factors, mitogenic hormones, metabolic inputs, and perceived physical signals from cell microenvironment, suggesting its crucial role in normal physiology. Moreover, Hippo signaling dysregulation has been linked to various human diseases like developmental anomalies, impaired immunity, cancer development, cancer metastasis and drug resistance. Therefore, elucidating the molecular basis of Hippo pathway regulation and underlying mechanism will not only provide novel insights into many fundamental processes in physiology, but also foster the development of therapeutic strategies for translational medicine. Here, we prepare a special Research Topic issue to provide an overview of the up-to-date research findings on this exciting and burgeoning filed.

While various intracellular and extracellular signals have been discovered to regulate the Hippo pathway, the current focus in the field is how these signals converge on the Hippo pathway for growth control and tissue homeostasis.

In this issue, Cai et al. reviewed how the Hippo pathway effectors YAP and TAZ are modulated under different mechanical cues. Given the fundamental roles of YAP and TAZ in mechanotransduction, the authors discussed the potential pathological roles of YAP/TAZ in several human diseases involving mechanical cues, such as pulmonary hypertension, atherosclerosis, cardiac hypertrophy, fibrosis, musculoskeletal disorder, and cancer.

Calcium (Ca^2+^) functions as an essential intracellular messenger in a number of cellular signaling events (Clapham, 2007). Wei and Li summarized the molecular mechanisms for the Ca^2+^-mediated regulation of the Hippo pathway, underscoring the important role of Ca^2+^-mediated actin reset (CaAR) in this process. Uncovering additional effectors for Ca^2+^ signaling with relevance to the Hippo pathway will enrich our understanding of the Hippo pathway regulation from a new perspective.

The tissue architecture-related cell polarity, cell-cell junctions and cell-extracellular matrix (ECM) interaction are known regulators of the Hippo pathway. Using *Drosophila* wing discs as a model, Wang et al. identified *Wallenda* (*Wnd*) (MAP3K13 in human) as a new player in the cell polarity-mediated Hippo pathway regulation. *Wnd* is involved in this process through nemo-like kinase, *Nmo* (NLK in human), and such *Wnd*-*Nmo* axis in regulating the Hippo pathway is shown conserved in evolution. Thus, it will be interesting to further examine whether dysregulation of these newly discovered regulators would cause cell polarity-associated human diseases like cancer.

Although the Hippo pathway kinase cascade transduces many upstream signaling events to YAP and TAZ, mechanisms that modulate YAP and TAZ activities independent of the Hippo kinase cascade do exist. In this Research Topic issue, Cho and Jiang authoritatively highlighted the recent findings of such “non-canonical” regulation for YAP and TAZ. These mechanisms highlight the complex regulation of the Hippo pathway, connect the Hippo pathway with other key cellular signaling events, and reveal novel therapeutic strategies for future investigations.

To resolve the complex regulation of the Hippo pathway, extensive efforts have been made to identify new Hippo pathway regulators. In line with it, Pipchuk and Yang described the application of luciferase-based biosensors to the Hippo pathway study. The authors discussed several assays using split luciferase complementation systems that have been successfully used for identifying new Hippo pathway regulators, highlighting the advantage of this luciferase-based biosensor method in studying real-time protein-protein interaction in live cells.

Although the Hippo pathway is known for its crucial role in organ size control, recent studies have extended it to other biological processes, such as embryogenesis, stem cell regulation and tissue regeneration. Here, Zhao et al. reviewed the emerging findings of the Hippo pathway in neural crest (NC) development. This work summarized the critical role of the Hippo pathway in many aspects of NC development, including NC initiation, migration, proliferation, survival and differentiation, as well as the NC-related diseases caused by the dysregulated Hippo signaling. In terms with organogenesis. Wu et al. described how the Hippo pathway acts in pancreatic development. This review highlighted the role of the Hippo pathway in progenitor cell maintenance and normal proper cell polarization/branching during pancreatic organogenesis and morphogenesis, which involve the crosstalk with several key developmental signaling pathways, such as Notch, Wnt, and PI3K-Akt.

Given the critical role of the Hippo pathway in numerous physiological processes, its dysregulation has been linked to many human diseases such as cancer. For example, the Hippo pathway is implicated to control cancer stem cell expansion and maintenance against cancer development (Park et al., 2018). To reveal the underlying mechanisms, Shen et al. investigated the Hippo pathway effector TAZ in breast cancer stem cells (BCSCs) and revealed the Cyclin D1-CDK4/CDK6 axis as downstream effector for the TAZ-driven breast tumorigenesis. This work suggests a possible vulnerability for BCSCs as well as a new opportunity for breast cancer therapy.

As for the cancer-related Hippo pathway dysregulation, He et al. systematically described the mutation and copy number abnormality for the Hippo pathway components in human cancer genome. Although the alterations of major Hippo pathway components in human cancers are rare, the impaired Hippo pathway may arise from the regulators of its core kinase cascade and/or nuclear transcriptional complex.

By examining The Cancer Genome Atlas (TCGA), Gu et al. analyzed the Hippo pathway kinase *LATS2* in the isocitrate dehydrogenase1/2 (IDH1/2)-mutated low-grade glioma (LGG). Their findings confirmed the hypermethylation of *LATS2* promoter in IDH1/2-mutated LGG but failed to link this genomic alteration with YAP activation experimentally. Thus, the role of the *LATS2* hypermethylation in LGG development deserves further investigation.

Studies from multiple model systems have fully established the Hippo pathway as an evolutionarily conserved signaling cascade from unicellular organisms to humans (Sebe-Pedros et al., 2012). In the past years, tremendous efforts have been made to elucidate the mechanisms for the Hippo pathway regulation and function, providing a reliable molecular basis for further exploring diverse biological processes and human diseases. In this Research Topic issue, the 10 articles written by leading scientists in the field cover the updated research findings for the Hippo pathway from different aspects. Advances in biotechnology including gene editing and single-cell studies are in great progress to expand our insights into the nature of Hippo signaling, especially from the perspectives of cell-cell interaction and organ-organ communication. We anticipate more discoveries of this exciting pathway will be made in years to come, allowing us to unveil the mystery of organ size control and develop new approaches for treating human diseases.

## Author Contributions

ZZ, ZY, WH, and WW wrote the Editorial. All authors contributed to the article and approved the submitted version.

## Conflict of Interest

The authors declare that the research was conducted in the absence of any commercial or financial relationships that could be construed as a potential conflict of interest.

## Publisher's Note

All claims expressed in this article are solely those of the authors and do not necessarily represent those of their affiliated organizations, or those of the publisher, the editors and the reviewers. Any product that may be evaluated in this article, or claim that may be made by its manufacturer, is not guaranteed or endorsed by the publisher.
